# Removal of phenol and bisphenol-A catalyzed by laccase in aqueous solution

**DOI:** 10.1186/2052-336X-12-93

**Published:** 2014-06-11

**Authors:** Zahra Asadgol, Hamid Forootanfar, Shahla Rezaei, Amir Hossein Mahvi, Mohammad Ali Faramarzi

**Affiliations:** 1 Department of Pharmaceutical Biotechnology, Faculty of Pharmacy and Biotechnology Research Center, Tehran University of Medical Sciences, P.O. Box 14155–6451, Tehran 14176, Iran; 2 Department of Environmental Health Engineering, School of Public Health, Tehran University of Medical Sciences, Tehran, Iran; 3 Department of Pharmaceutical Biotechnology, Faculty of Pharmacy, Kerman University of Medical Sciences, Kerman, Iran; 4 Center for Water Quality Research, Institute for Environmental Research, Tehran University of Medical Sciences, Tehran, Iran; 5 Center for Solid Waste Research, Institute for Environmental Research, Tehran University of Medical Sciences, Tehran, Iran

**Keywords:** Removal, Laccase, *Paraconiothyrium variabile*, Pollutant, Bisphenol A

## Abstract

**Background:**

Elimination of hazardous phenolic compounds using laccases has gained attention during recent decades. The present study was designed to evaluate the ability of the purified laccase from *Paraconiothyrium variabile* (*Pv*L) for elimination of phenol and the endocrine disrupting chemical bisphenol A. Effect of laccase activity, pH, and temperature on the enzymatic removal of the mentioned pollutants were also investigated.

**Results:**

After 30 min treatment of the applied phenolic pollutants in the presence of *Pv*L (5 U/mL), 80% of phenol and 59.7% of bisphenol A was removed. Increasing of laccase activity enhanced the removal percentage of both pollutants. The acidic pH of 5 was found to be the best pH for elimination of both phenol and bisphenol A. Increasing of reaction temperature up to 50°C enhanced the removal percentage of phenol and bisphenol A to 96.3% and 88.3%, respectively.

**Conclusions:**

To sum up, the present work introduced the purified laccase of *P. variabile* as an efficient biocatalyst for removal of one of the most hazardous endocrine disruptor bisphenol A.

## Introduction

Phenol is one of the most environmentally hazardous pollutants which increasingly been applied by industries like petrochemical, coking, plastics, paper and oil refineries, as well as phenolic resin industries [1,2]. Poor solubility and biodegradability, long term ecological damage and high toxicity of phenol and its derivatives led to calls by Environmental Protection Agency (EPA) for lowering phenol concentration in the wastewater to less than 1 mg/mL before discharging them into the water reservoirs [3]. The estrogenic action and impacts of the bisphenol A (4,4′-isopropylidenediphenol, BPA), an industrial chemical which is widely used for the synthesis of polycarbonate plastics and epoxy resins, on female reproductive tract introduced BPA as an endocrine disrupting chemical (EDC) [4,5]. The role of BPA exposure in development of prostate and breast cancers, reduction of human sperm counts, alteration of immune functions, prevalence of obesity and decrease fertility in fish and mammals has been demonstrated by some studies [5,6]. So, various physicochemical processes like activated carbon adsorption, solvent extraction, chemical oxidation and electrochemical methods have been developed to remove phenolic compounds from wastewaters [5,7-13]. However, wide applications of some of these remediation strategies have been limited due to problems such as high cost, low efficiency, and generation of toxic by-products [14,15]. On the other hand, advantages of biological techniques including biodegradation of xenobiotics using living microorganisms like algae [16], bacteria and fungi and/or their purified oxidizing enzymes both in free [17,18] and immobilized form [19-22] as well as biosorption of organic pollutants [1,23] introduced this field as a novel area for removal of hazardous compounds.

Laccases (benzenediol: oxygen oxidoreductase, EC 1.10.3.2) are copper containing oxidases catalyzing the oxidation of a wide range of aromatic substrates including phenol derivatives, benzenethiols, polyphenols [24] and polycyclic aromatic hydrocarbons (PAHs) [25]. In recent decades, laccase-producing microorganisms, especially white-rot fungi [25,26] have been employed for biological treatment of different pollutants [27]. Biodegradation of brominated phenols using cultures and laccase of *Trametes versicolor* was investigated by Uhnakova et al. [26]. Zhang et al. [24] studied on degradation of 2,4-dichlorophenol (2,4-DCP), 4-chlorophenol (ρ-CP), and 2-chlorophenol catalyzed by laccase from *Coriolus versicolor*.

The aim of the present study was to investigate on the ability of the purified laccase from the ascomycete *Paraconiothyrium variabile*, a newly isolated laccase-producing ascomycete from soil, for elimination of phenol and bisphenol A. Effect of parameters such as laccase activity, pH, and temperature on removal of pollutants was also studied.

## Materials and methods

### Laccase and chemicals

The produced laccase of *P. variabile* was purified from fungal culture broth based on the method described by Forootanfar et al. [28] and applied in pollutant removal experiments. Phenol, bisphenol A (BPA), and 4-aminoantipyrine (4-AAP) were purchased from Merck (Darmstadt, Germany). 2,2′-Azinobis-(3-ethylbenzthiazoline-6-sulphonate) (ABTS) was obtained from Sigma-Aldrich (St. Louis, MO, USA). All other chemicals were of analytical grade.

### Determination of laccase activity

Oxidation of ABTS as a laccase substrate was used to determine the laccase activity [29,30]. In brief, 0.5 mL of enzyme sample was added to 0.5 mL of ABTS solution (5 mM in 0.1 M citrate buffer, pH 4.5) and incubated at 37°C and 120 rpm for 10 min. Change in absorbance at 420 nm was monitored by a UV/vis spectrophotometer (UVD 2950, Labomed, Culver City, USA) and the laccase activity was calculated using the molar extinction coefficient of ABTS (ϵ_420_ = 36,000 M^-1^ cm^-1^). One unit of laccase activity was defined as the amount of enzyme required to oxidize 1 μmol of substrate per minute [31].

### Removal studies

In order to study on the ability of the purified laccase for elimination of phenolic pollutants, the reaction mixture (final volume of 3 mL) was prepared as follow: 1 mL of phenol or BPA solution (final concentration of 4 mM) was added to 1 mL citrate buffer (20 mM pH 5) followed by introducing of the purified laccase (final concentration of 5 U/mL) to the reaction mixture and incubation at 35°C and 50 rpm for 40 min. Samples were taken every 10 min and analyzed for remaining concentration of phenolic pollutants. The negative control was designed by inserting of heat-inactivated laccase to the reaction mixture. Each experiment was performed in triplicate and mean of the obtained results were reported.

### Determination of phenol and bisphenol A concentration

Concentration of phenolic pollutants was measured using a colorimetric assay in presence of 4-AAP as a primary amine [6]. Briefly, the reaction mixture was prepared by addition of 700 μL of phosphate buffer (0.1 M pH 8), 300 μL of laccase-treated phenol or BPA sample, 10 μL of 4-AAP (0.1 M) and 10 μL potassium ferricyanide solution (0.2 M) followed by incubation at 25°C and 100 rpm for 15 min. The absorbance of the reaction mixture was then measured at 506 nm and the phenol or BPA concentration was determined from the obtained standard curve.

### Effect of laccase activity on the removal of phenolic pollutants

The effect of laccase activity on the removal of phenol or BPA was studied by adding of enzyme solution (1, 5, 10 and 20 U/mL in citrate buffer 20 mM pH 5) to the phenolic pollutant solutions (final concentration of 4 mM) and incubation at 35°C and 50 rpm for 30 min. The reaction mixture was then analyzed for remained phenolic pollutant concentration as described above.

### The effect of pH on laccase-mediated removal

After adjusting the initial pH of the phenolic pollutant solution (final concentration of 4 mM) using 20 mM citrate-phosphate buffer between 3–7, the purified laccase (5 U/mL) was added to the reaction mixture and incubated at 35°C and 50 rpm for 30 min. The pollutant concentration was then monitored as previously described.

### The influence of temperature on enzymatic removal

The effect of temperature on enzymatic elimination of pollutants was studied by incubating 4 mM of the phenolic pollutant solution (in citrate buffer 20 mM pH 5) in the presence of laccase (5 U/mL) at temperature range of 30–70°C.

## Results and discussion

### Pollutant elimination by the purified laccase

**Figure 1:**
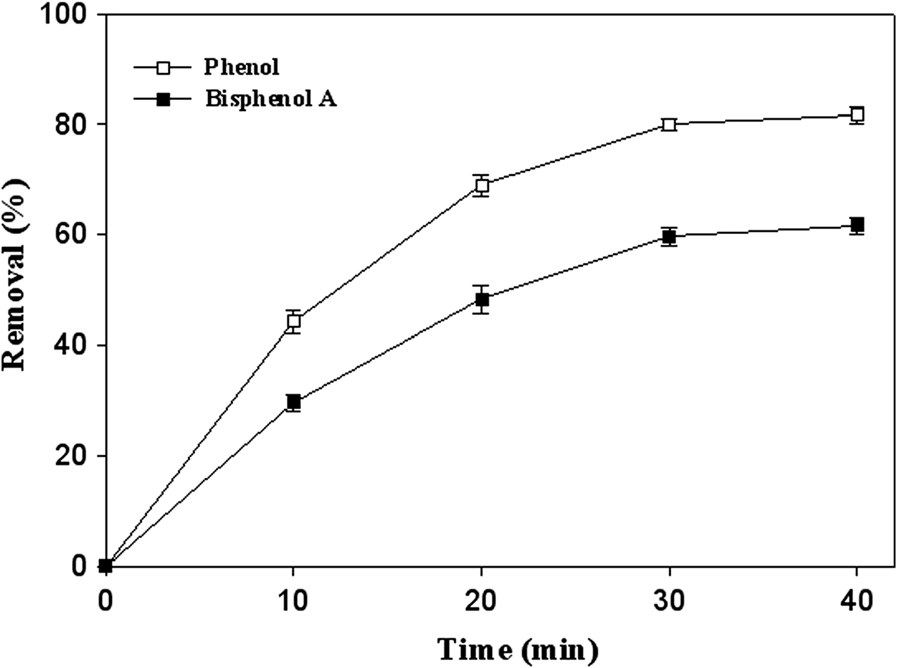
**Time courses of phenol and bisphenol A removal (initial concentration of 4 mM) using the purified laccase of *P. variabile *** **(5 U/mL) dissolved in citrate buffer 20 mM pH 5.**

As shown in the time course of phenolic pollutant elimination curves (Figure 1) the purified *Pv*L removed 29.6% and 44.3% of BPA and phenol, respectively after 10 min treatment. After 30 min, the removal percentages increased by 59.7% and 80% in the case of BPA and phenol, respectively (Figure 1). No elimination was detected in the case of negative controls. The enzymatic removal of phenol and related hazardous compounds especially the endocrine disruptor compound (bisphenol A) discharged from industrial effluents into the environment has received more attention during recent decades [32,33]. Potential of *Pv*L for removing of a wide range of chemicals including chlorophenols, synthetic dyes and benzodiazepines have been shown in recent studies [14,15,22,27]. The present study showed that both applied phenolic pollutants (initial concentration of 4 mM) was eliminated using the purified laccase of *P. variabile* after 30 min incubation. Kurniawati et al. [33] reported about removal of phenol (initial concentration of 500 μM) after 6 h incubation in presence of the purified laccase from *Trametes versicolor*. The purified laccase of *Fusarium incarnatum* UC-14 hosted in the reversed micelle was able to remove 91.43% of bisphenol A (200 ppm) after 2 h incubation [5].

### The effect of laccase activity on removal of phenolic pollutants

**Figure 2:**
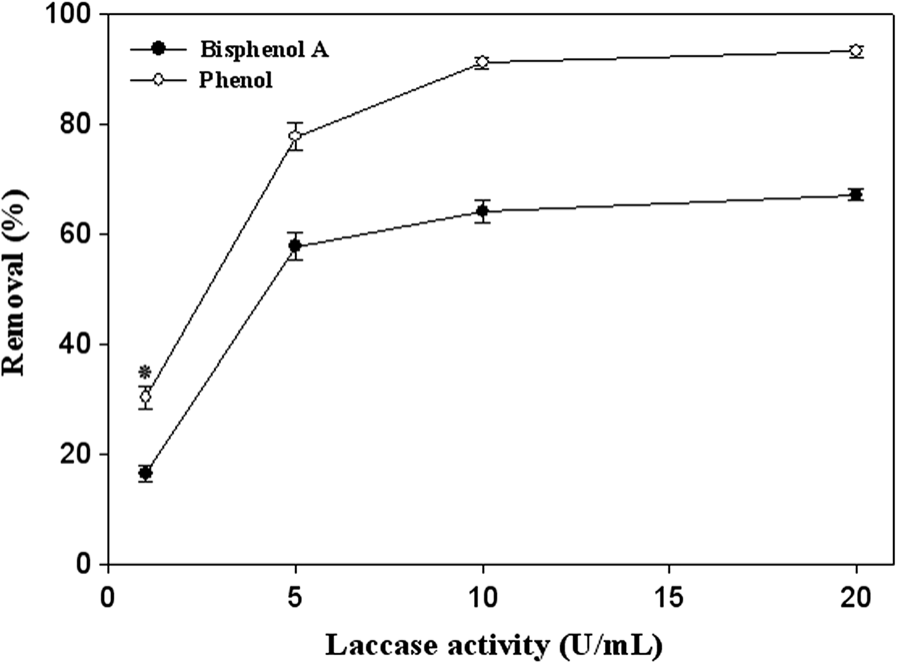
Effect of laccase activity on removal percentage of phenolic pollutants (initial concentration of 4 mM) after 30 min enzymatic treatment.

The pattern of removal of pollutants by increasing of laccase activity from 1 U/mL to 20 U/mL is illustrated in Figure 2. In the present study, it was showed that elimination of both phenol and bisphenol A increased when laccase activity was enhanced. Same results was observed in study of Okazaki et al. [34], which indicated that increasing of laccase concentration (originated from *Coriolus versicolor*) from 0 to 50 μg/mL enhanced BPA removal to 100%. Application of the purified laccase from *Trichoderma atroviride* (0.3 U/mL) for elimination of phenolic pollutants including 2,4-dichlorophenoxyacetic acid, 4-chlorophenol, o-cresol or and catechol led to 21%, 28%, 100% and 100% removal of pollutants, respectively after 24 h incubation [35].

### The influence of pH on pollutant removal

**Figure 3:**
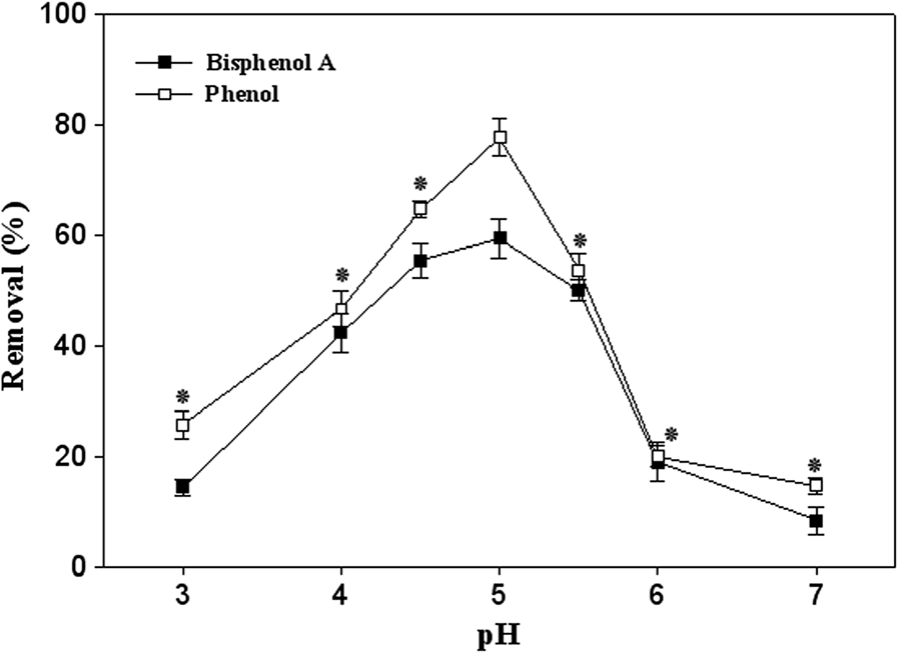
**Influence of pH alteration on removal of bisphenol A and phenol in presence of the purified laccase of *P. variabile *** **(5 U/mL) after 30 min enzymatic treatment.**

As illustrated in Figure 3, both of studied pollutants represented maximum removal percentages (77.7% and 59.3% in the case of phenol and BPA, respectively) at pH 5, which was previously introduced as the optimum pH for the activity of the purified laccase from *P. variabile*[28]. Majority of the fungal laccases optimally act in acidic pH [36]. In the study of Chhaya and Gupte [5], who evaluated the activity of laccase from *Fusarium incarnatum* UC-14 toward BPA, the pH value of 6 was introduced as optimal pH for removal of bisphenol A. study of Liu et al. [37] showed that acidic environment (pH 6) was the best condition for phenol removal using the recombinant laccase of *T. versicolor*.

### The effect of temperature on laccase-mediated pollutant removal

**Figure 4:**
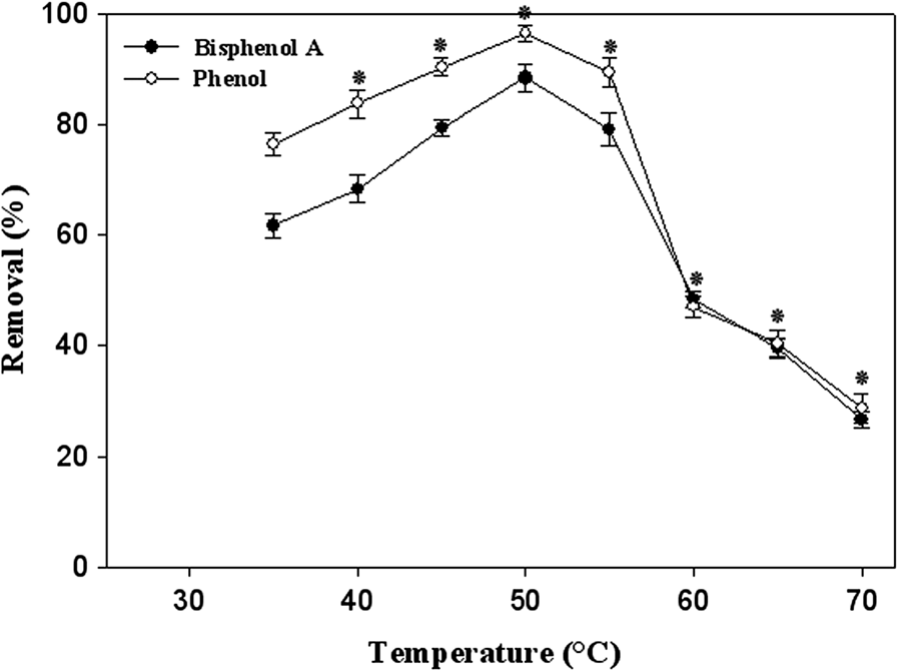
**Effect of temperature on bisphenol A and phenol removal after 30 min treatment by the purified laccase of *P. variabile *** **(5 U/mL).**

The optimum temperature for the elimination of phenol (96.3% removal) and BPA (88.3% removal) was found to be 50°C (Figure 4). This temperature is the optimum temperature of *Pv*L activity determined by Forootanfar et al. [28]. At elevated temperature of 60°C the removal percentage of phenol and BPA in presence of *Pv*L dropped to 47% and 48.3%, respectively. These results were in agreement with the findings of Kurniawati and Nicell [33], who determined maximum laccase-assisted phenol elimination between 40°C and 50°C and a sharp decrease in pollutant removal was observed above 60°C. In the study of Kim et al. [4] maximum of bisphenol A degradation (67%) using the laccase of *Trametes versicolor* (0.15 U/mL) was achieved at temperature of 45°C. Most of the fungal laccases maximally act in the temperature range of 50–70°C, although the maximum activity of laccase from *G. lucidum* was at 25°C [36].

## Conclusion

The purified laccase of *P. variabile* applied for removal of phenol and bisphenol A. *Pv*L was efficiently eliminated both applied pollutants after 30 min treatment. Maximum of removal percent in the case both phenolic pollutants was obtained at optimum pH and temperature of the *Pv*L. To sum up, the results of the present investigation candidate the purified *Pv*L for removal of phenolic pollutants. However, more study should be conducted to find out about probable produced metabolites.

## Competing interests

The authors declare that they have no competing interests.

## Authors’ contributions

ZA carried out the elimination studies of phenol and bisphenol A. Production and purification of laccase from *P. variabile* culture broth was performed by SR and HF, respectively. AHM contributed in writing of the manuscript, elimination studies and analyzing of data. MAF involved in purchasing of required materials and instruments, designing of removal experiments, analyzing of data and reviewing of the manuscript. All authors read and approved the final manuscript.
